# Using digitally mediated methods in sensitive contexts: a threat analysis and critical reflection on data security, privacy, and ethical concerns in the case of Afghanistan

**DOI:** 10.1007/s42597-022-00088-2

**Published:** 2023-01-10

**Authors:** Laura Gianna Guntrum, Benjamin Güldenring, Franz Kuntke, Christian Reuter

**Affiliations:** 1 Science and Technology for Peace and Security (PEASEC), Department of Computer Science, Technical University of Darmstadt, Pankratiusstraße 2, 64289 Darmstadt, Germany; 2 Secure Identity, Department of Computer Science, Freie Universität Berlin, Schwendenerstraße 1, 14195 Berlin, Germany; 3 Reporters Without Borders (RSF), Berlin, Germany

**Keywords:** Digital research design, Research ethics, IT security, Secure communication, Technical peace and conflict research, Threat modeling, Digitales Forschungsdesign, Forschungsethik, IT-Sicherheit, Sichere Kommunikation, Technische Friedens- und Konfliktforschung, Bedrohungsmodellierung

## Abstract

Given the lack of empirical examples of how research can be conducted via digital means in sensitive contexts, this paper provides a threat model using Afghanistan, where the Taliban took power in August 2021, as an example. Both technical and non-technical research-related risks are analyzed, paying attention to research ethics, data security, and privacy. We argue that any threat model and risk analysis is highly context-dependent. Our analysis reveals that in certain research processes, human security does not necessarily coincide with data security and that an ambivalence exists between privacy and usability. In addition to the concrete threat analysis, the paper identifies some general technical solutions (e.g., encryption methods, communication software) for different research steps to foster secure and ethically justifiable research.

## Abstract (de)

Mangels empirischer Beispiele dafür, wie Forschung digital in sensiblen Kontexten durchgeführt werden kann, wird in diesem Beitrag ein Bedrohungsmodell am Beispiel Afghanistans vorgestellt, wo die Taliban im August 2021 die Macht übernommen haben. Es werden sowohl technische als auch nichttechnische forschungsbezogene Risiken analysiert, mit Fokus auf Forschungsethik, Datensicherheit und dem Schutz der Privatsphäre. Wir argumentieren, dass jedes Bedrohungsmodell und jede Risikoanalyse stark kontextabhängig ist. Unsere Analyse zeigt, dass in bestimmten Forschungsprozessen die Sicherheit der Menschen nicht unbedingt mit der Datensicherheit übereinstimmt und dass eine Ambivalenz zwischen Privatsphäre und Nutzbarkeit besteht. Neben der konkreten Bedrohungsanalyse werden im Beitrag einige allgemeine technische Lösungen (z. B. Verschlüsselungsmethoden, Kommunikationssoftware) für verschiedene Forschungsschritte aufgezeigt, um eine sichere und ethisch vertretbare Forschung zu fördern.

## Communication software as an ethnographic tool

In recent years, communication software, especially instant messengers, social media, and video conference software, have become relevant for mutual quick and inexpensive everyday communication—also within academia (Tanczer et al. 2020). The COVID-19 pandemic has further highlighted our reliance on digital media to conduct cross-national studies in times of travel and contact restrictions (Lawrence 2022). Not least due to privacy violations, recent surveillance incidents (for example regarding academic researchers and human rights activists in El Salvador affected by the Pegasus spyware), and arrests as a result of the inspection of social media accounts by authoritarian actors (such as during the protests in Myanmar in 2021), awareness about potential risks associated with the use of digital applications is slowly gaining importance among different parties (Grimm et al. 2020; Potkin and Mcpherson 2021; Jones 2022). Most recently, there has been growing concern about Afghanistan, where the Taliban took power in August 2021. There is strong indication that the Islamic fundamentalist group will increasingly monitor online activities (Tangen 2021), potentially resulting in devastating consequences for the Afghan population. In case digital research is to be conducted in such unstable contexts, numerous ethical and data security aspects need to be considered before, during, and after conducting research, following the principles of research ethics (Hammersley and Traianou 2012; Lauber-Rönsberg 2018). Generally, “technologically supported censorship and surveillance impinge upon scholars’ ability to conduct unobstructed inquiry” (Tanczer et al. 2016, p. 346) and negative consequences, such as data misuse and digital surveillance, which may be related to research, indicate how important it is to pay sufficient attention to digital security in order to protect involved stakeholders in times where physical and digital spaces overlap.

In any research via digital means, the question should be asked whether risks (e.g., digital surveillance) are likely and whether more data secure alternative applications could be used during the entire research process, including data analysis and storage, since multiple programs (e.g., for transcription) raise privacy concerns. In software engineering, for example, the process of threat modeling is established to identify computer security threats in a proactive manner (Xiong and Lagerström 2019). Researchers in other fields should also use similar techniques in advance of conducting any study to identify most likely attack vectors and potential adversaries. Particularly in hostile environments, theoretical approaches from peace and conflict studies, including ethical research in conflict contexts and the concept of *do-no-harm*, are equally useful and important (Wood 2006; Moss et al. 2019). Consequently, valuable approaches from different disciplines should complement each other in order to provide a holistic analysis (Quinton and Reynolds 2018). Currently, however, there is a lack of empirical examples in the academic literature that illustrate how theoretical approaches from peace and conflict research and threat modeling can be applied in practice. Therefore, this paper aims to make an important contribution to existing academic discussions by exemplarily presenting a hypothetical case study on Afghanistan (2021), in which online interviews with Afghan journalists are planned. Overall, the paper provides thought-provoking information and aims to raise awareness about privacy, the data security it requires, and ethical concerns. However, it should not be seen as a universal guide since risks and recommendations may differ significantly and are very time and context specific. Rather, the paper aims to be a contribution to the concrete application of theoretical concepts by combining approaches from both the social sciences and computer sciences. This integration is particularly relevant against the backdrop of the fast pace of technological development and constantly changing contexts.

Inspired by these research gaps, we formulated the following research question (RQ): *How can a threat model generally be elaborated in order to identify potential risks and adversaries in sensitive context, having privacy, security, and ethical concerns in mind? *The following two sub-questions specify the RQ, relate to the case study, and illustrate the practical procedure of a threat model as well as its practical implications: 1) What could a threat model for Afghanistan look like, considering the situation after the Taliban took power in 2021? and 2) Which existing information and communication technology is suitable for digital research in sensitive contexts such as Afghanistan? Aiming to answer the formulated research questions and to empirically apply theoretical concepts, we will address individual research steps, such as data collection and data storage. We aim to investigate which technical options (e.g., encryption) are perceived to be relevant for each individual step.

In the following, we present a literature review of previous studies related to ethics in digital research and information technology (IT) security (section “Related work and theoretical embedding”) and explain our research design in detail (“Methodology” section). Drawing on a more general description of threat modeling, we present a threat model on the case of Afghanistan in the section “Hypothetical case study: Afghanistan (2021)”. Subsequently, based on existing categories from IT security for secure and privacy-compliant communication, we examine which communication tools appear suitable for conducting secure digitally mediated research, having the empirical study of Afghanistan in mind (section “What to consider when working with digitally mediated methods”). Finally, we discuss the results (section “Discussion and conclusion: secure research with digitally mediated methods”) and address some limitations of our work (section “Limitations”).

## Related work and theoretical embedding

In the following, we present key literature on 1) research ethics in sensitive contexts, 2) research ethics in the digital age, and 3) threat modeling. We consider these concepts essential for understanding our empirical case study, as we aim to apply existing theoretical concepts from different disciplines to a concrete case. Overall, we would like to emphasize that we only focus on the literature that we consider essential to answer our RQs.

### Research ethics in times of digitization

In general, research related risks such as psychological stress, traumatization, arrest, and distrust should be minimized for all individuals engaged in a research process (Fujii 2012; Moss et al. 2019). Adherence to ethical principles helps to mitigate these risks and should be an “ongoing responsibility” for everyone, according to Fujii (2012). Commonly, “conducting research ethically means to do research in a safe and secure manner where participants’ wellbeing and interests are safeguarded during the pursuit of knowledge” (Tolich and Tumilty 2021, p. 1). This means first and foremost that “individuals should be treated as autonomous beings, capable of making their own decisions” (Markham and Buchanan 2015, p. 607). Here, approaches from the Menlo Report (2012) such as justice, beneficence, respect for persons, the Belmont Report (1979), and concepts such as do-no-harm should be considered, incorporating conflict sensitivity wherever it is of relevance (Anderson 1999; Kenneally and Dittrich 2014; Baele et al. 2018). Scientists conducting research need to be aware that unethical research betrays participants’ trust, possibly endangers their lives, and hampers future empirical research, as people might reject scientific studies due to bad experiences (Lauber-Rönsberg 2018; Barbosa and Milan 2019). According to Tanczer et al. (2020, p. 3), “new challenges that information and communication technologies have brought to the scholarly profession” are not widely discussed.

Generally, safety-related aspects are particularly relevant in sensitive research contexts. Regarding research in sensitive contexts, Cohen and Arieli (2011) emphasize that “methodological aspects of field work in conflict environments have not been systematically analyzed” and that various (methodological) challenges, such as identifying respondents’ needs and cultural differences, might complicate research processes. The consequence of these challenges is that marginalized individuals as well as countries affected by crisis are least investigated with respect to “in-depth field research” (Clark 2006). This status quo is changing slowly, partly because technical achievements and digitally mediated methods facilitate participation of historically marginalized groups and provide access to research contexts, which were previously difficult to reach because of security issues or travel restrictions (Chiumento et al. 2018). Overall, remote research is becoming increasingly common these days for numerous reasons, including being more environmentally friendly, saving costs and time, as well as contacting people in hard-to-reach areas and situations (Williams 2012; Dawson 2020), and “building more equitable collaborations” (Mwambari et al. 2021, p. 2). Furthermore, our own previous research experiences have shown that digital research may be more secure than physical research in some cases (if certain considerations are taken into account), as the presence of (*white*) researchers in certain areas may generate attention and skepticism. Obviously, digitally mediated research also entails disadvantages and challenges. Barbosa and Milan (2019, p. 50), for example, examine “ethical challenges of conducting research of an ethnographic nature on WhatsApp.” Furthermore, disadvantages include impeded nonverbal communication and trust-building, a lack of physical proximity, digital surveillance, and new dimensions of privacy violations—especially when personal or biometric data is collected (Venter 2019; Mwambari et al. 2021; Troncoso 2021).

According to Dobrick et al. (2018), to date little research has been done on privacy violations and data security^1^ in social sciences, including peace and conflict studies. This seems astonishing, since numerous empirical studies in peace and conflict studies have collected sensitive data that can potentially be captured or be under surveillance by adversarial actors—such as the military, authoritarian regimes, or state actors in general—who would likely misuse collected data and might harm involved parties. Peace and conflict researchers in particular should pay special attention to better understand and address major societal challenges, as this research community emphasizes the importance of collecting data, especially in sensitive contexts (Wissenschaftsrat 2019).

Concerning ethics in digital research, the state of the literature demonstrates that particularly the health sciences have addressed ethical issues related to digitally mediated research designs (Yip et al. 2016). According to Markham and Buchanan (2015, p. 606), “ethical questions [involving the internet and digital materials] have focused for many years on areas such as representation, privacy, the nature of public data and identities.” Quinton and Reynolds (2018, p. 21) state that “many aspects of the digital environment complicate ethics questions and decisions that are well-understood in the non-digital context.” Against this background, and taking into account that to date there are no definitive responses to the existing challenges in the field of digital research, approaches from the field of Internet Research Ethics and guidelines from organizations such as the Association for Computing Machinery (ACM) or Electronic Frontier Foundation (EFF) may be used as guiding principles, even though they are optional and do not provide enforcement mechanisms in case of violation (IEEE 2001; Buchanan and Ess 2009; Association for Computing Machinery 2018; Barbosa and Milan 2019). This illustrates the difficulty in implementing a binding guideline for ethical behavior in the context of digital research and the need “to formulate appropriate rules for scientific research” (Lauber-Rönsberg 2018, p. 42) within one’s own research discipline.

In this regard, the discipline of IT security becomes increasingly important. Already since the 1990s, data protection patterns such as encryption and threat modeling have been developed to mitigate existing risks, although “they have been plagued by usability issues” (Unger et al. 2015, p. 232). So far, several studies—amongst others Abu-Salma’s et al. (2017)—have examined IT security aspects in sensitive contexts. Others have looked at the usability of secure communication tools, comparing them in terms of privacy and data security (Unger et al. 2015; Ermoshina et al. 2017; Aggarwal et al. 2018; Electronic Frontier Foundation 2018). While communications software that prioritizes privacy and security exist, relatively few people use it on a global scale. Studies indicate that the vast majority of people use applications that are utilized by a large number of their close environment, without regard to existing vulnerabilities (De Luca et al. 2016). Moreover, most applications have certain constraints, which is why trade-off usually exists between usability, security, and distribution. This seems to be the case not only for communication tools but also for other applications developed to provide privacy, such as password managers (Oesch and Ruoti 2020).

In summary, operationalizing established policies and strategies in traditional analog research for digitally mediated research designs is a fundamental challenge (Sugiura et al. 2017). Comprehensive risk assessment has proven difficult for many scientists, since theoretical concepts are often highly abstract and certain aspects, such as surveillance, occur secretly and invisibly. Additionally, many researchers do not know how to counteract potential threats as they lack domain expertise (Moßbrucker 2020). In such cases, it might be helpful to study existing security guides for digital research (AccessNow 2022; Front Line Defenders 2021) or to seek online support from IT security experts, who are specialized in technical support for human rights activists and journalists in conflict-related contexts (Digital First Aid Kid 2022). In conclusion, it shall be noted that “when making ethical decisions, researchers must balance the rights of subjects (…) with the social benefits of research and researcher’s right to conduct research” (Markham and Buchanan 2015, p. 607).

### Security plan and threat modeling

Particularly in sensitive contexts, as already mentioned in the previous section, researchers must evaluate potential direct or indirect threats (Kazansky 2021). According to Jeong (2008), “multidimensional frameworks of analysis are necessary to examine conflicts comprising diverse types of parties and issues.” For this purpose, the EFF (2021a) developed a security plan for digital information to guide research projects to “understand the unique threats you face and how you can counter these threats.” The security plan addresses questions about potential risks and consequences that might help to assess which risks may exist in one’s own study. Prior to an empirical study, data mapping and actor mapping should be conducted, i.e., an overview about what data will be collected and an analysis of actors, who might have an interest in the (personal) data obtained, but who should not gain access. In IT security, these actors are typically referred to as adversaries. Typical profiles of adversaries are classified based on their level of abilities (skills), motivation/maliciousness, and incorporated methods (Meyers et al. 2009). Examples of adversary profiles are nation-states, the military, or terrorists. However, depending on the research context and the specific individuals involved in the research process, researchers should not only consider IT-centric adversary profiles. In addition, risks posed by the immediate research environment should also be considered, e.g., with regard to the study participants’ family members (Vashistha et al. 2018) as well as other real-world contacts, such as police officers or militants, that may interact with participants or researchers. Henry et al. (2022) emphasize risks, especially during the initial phase and after the end of a project and give a (high-level) checklist to guide researchers in assessing risk by asking appropriate questions for each phase of a research project. However, we see an additional need to better support researchers in choosing secure tools and methods during the conduct of studies, in addition to the important aspect of potential misuse of study results after publication.

In general, the most important principle of any research project should be to ensure human security. However, other ethical aspects shall also be considered, for example, that people are not forced into situations that they do not want. Such undesirable situations also exist in the digital realm, for example, when people are forced to use certain software that compromises their data security and may thus violate their right to privacy. On the other hand, sometimes less data-secure tools that violate privacy principles may be an appropriate option when conducting research in sensitive contexts—for example, if such tools, unlike their more privacy-friendly alternatives, do not raise suspicion during identity checks, physical harm can be better prevented. Choosing the right tools and methods require to detect and analyze potential threats. Grimm et al. (2020b) present a digital threat modeling guideline for identifying data-related threats and assessing their likelihood. This contains three main aspects including the following guiding questions (Grimm et al. 2020b):

1. Data mapping: What types of data do you produce or make use of during your research? What information do you collect, store, or communicate? What types of contextual data or metadata are being created throughout the research process? What would be the consequences if either of these data types were compromised?
2. Actor mapping: Who might be interested in your data, the sources of your data, and in its further use? Who might obtain access to your communication via physical or digital means? How do these actors view your research project? By whom could your data be misused? What are their capabilities and how likely is it that they will use them? Which data are they most likely to use? Who would be affected by data breaches?
3. Context analysis: How accessible and reliable is the communication infrastructure? What software and ICT services do you rely on during your research? Does the legal context prohibit or prescribe the use of certain ICT services? What level of digital proficiency do you and/or your research team possess? Who else might have access to your computer?

Additionally, we argue for introducing a validation step, similar to traditional IT-based threat modeling:

4. Validation: Have you acted on each of the previous steps and answered all questions?

**Fig. 1:**
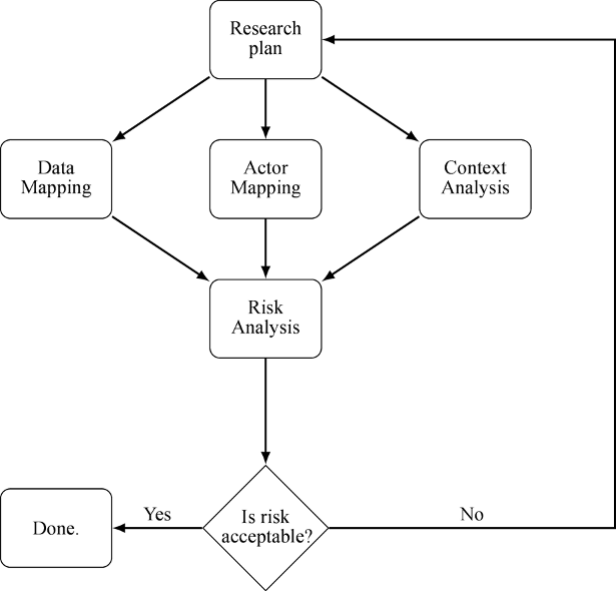
Iterative process in devising a research plan. (Source: own representation)

By answering these guiding questions, a matrix of potential threats can be created, together with their likelihood and impact. The more precisely such a threat matrix is filled, the more accurately one can estimate associated risks and the scope of necessary countermeasures (Grimm et al. 2020a). Appropriate countermeasures must be taken for all parts of research, starting with securing communication, which we have identified as the key part of a research project. From a practical perspective, we suggest an iterative process in devising a research plan. After an initial plan is made, data mapping, actor mapping, and context analysis feed the risk analysis step. The research plan is then refined until the remaining risk is deemed acceptable. Fig. 1 illustrates this process.

## Methodology

After having introduced some theoretical concepts, the methodological procedure will be briefly described.

### Case selection

As highlighted earlier, the academic literature is lacking empirical studies involving threat models and risk analyses. Therefore, we would like to fill this research gap with a concrete example. To explain the procedure of mapping security issues in a more practical way, we have chosen Afghanistan as a hypothetical scenario for an empirical investigation. Between 1996 and 2001, Afghanistan was ruled by the Islamic fundamentalist group the Taliban, which was overthrown by US-led forces after the attack on the World Trade Center. In 2021, after having regrouped in Pakistan, the Taliban returned to power subsequent to the withdrawal of international, particularly US, forces and eventually forced the government to resign. Building on experiences from times when the Taliban ruled the country according to Sharia law, “medieval methods and strategies” are to be expected to return, endangering the Afghan society (Modebadze 2022, p. 78). Besides the continuously deteriorating humanitarian and economic crisis, personal threats due to individual character traits (e.g., gender, occupation) or activities are increasing. Although the Taliban promised amnesty to Afghans working with foreigners and the protection of, among others, religious minorities, retaliatory killings and violence have been recorded. Due to its topicality and the complexity of violent incidents, Afghanistan is an important case for analysis in the context of peace and conflict studies.

### Data collection

In an attempt to understand the complexity of the research context, an embedded mixed-methods design was used.

#### Literature and internet research

For our threat model for Afghanistan, we refer to two sources: The first is the Digital Care Guide for Afghan journalists, produced by the non-governmental organization *Reporters Without Borders* (RSF) in 2021 to provide Afghan journalists with digital security advice (Reporters Without Borders 2022). The report is based mainly on qualitative interviews with Afghan journalists. RSF kindly provided us with early access to this guide. For the purpose of this paper we only refer to the publicly available information in this Digital Care Guide.^2^ Our second source is anecdotal evidence from our own work with Afghan journalists, our work in IT security and peacebuilding, our practical experiences in conflict regions, and contact with organizations operating on the ground.

In the following, concrete recommendations for action for the Afghan hypothetical case scenario shall be formulated, whereby it shall be shown that some of these recommendations may not be applicable to other research contexts. For this purpose, an in-depth literature search, including IT security blog posts and websites of organizations and foundations dealing with our research interest was conducted. Since little information is available in academic literature, the systematic search was extended to grey literature. In both academic journals and websites, search queries used for filtering include “research ethics,” “digital research,” and “secure communication.” Selected online contributions address both vulnerable individuals affected by human rights violations and people in contact with endangered individuals. In general, blog posts and organizations provide overall advice on data security, privacy, and usability that is comparatively comprehensive since people from different backgrounds have access to them and may contribute to different aspects. This diversity allows to gain insights from different perspectives.

#### Expert interviews

To gain more detailed and practice-oriented insight into how individuals collect data in sensitive contexts and how peacebuilding and IT security experts perceive existing communication software regarding data security, eleven (*N* = 11) interviews, were conducted between August and November 2021 (xmax = 62 min, xmin = 29 min, X̅ = 51 min). To facilitate the structuring of the interviews, the interviewers elaborated a semi-structured questionnaire, containing questions regarding challenges and opportunities related to digital research as well as concrete recommendations for applications. Considering that all interviewees are associated with either peace work (*n* = 6), academia (*n* = 3), app development (*n* = 1), or IT security (*n* = 1), they are considered experts in their respective field. Access to interviewees, coming from countries such as Iraq, Ukraine, and Afghanistan was facilitated by in-depth internet research (e.g., civil peace organizations, well-known IT security blogs) and recommendations (snowball sampling) (Moser and Korstjens 2018). A majority of people contacted were very interested in doing an encrypted interview via Signal, Zoom, Jitsi Meet (GDPR^3^ compliant), or to exchange their knowledge via eail. All respondents participated voluntarily, confirmed their consent, and were not financially compensated. Since the interviews dealt, inter alia, with their own data research procedures and sensitive information, all interviewees are pseudonymized and no personal information is disclosed.

The interviews are intended to complement given external recommendations on Afghanistan and personal experiences by suggesting other communication tools and strategies to conduct research in vulnerable contexts, highlighting that recommendations may vary depending on the specific circumstances.

### Analysis of data

Regarding our paper, it is important to address requirements and practices that are necessary for threat modeling and secure empirical studies in sensitive contexts. To define these requirements, the digital care guide, the literature, and the interviews conducted were analyzed by an interdisciplinary team, skilled in peace and conflict research and computer science. Regarding our own socioeconomic, educational, and cultural background (middle-class, *white*, cis-gendered European researchers), we tried to critically question our own perceptions, having the scientific quality criteria of objectivity in mind.

## Hypothetical case study: Afghanistan (2021)

In the following, we present a hypothetical scenario, closely resembling reality to better understand the procedure of a threat model. In this scenario, we imagine interviewing Afghan journalists via digital means. Note that interviews with different target groups in Afghanistan, such as human rights defenders or other civilians, might result in a similar assessment, but would incorporate a different discussion.

We begin our threat modeling with a short recap of the situation for journalists in Afghanistan in 2021. We then, as suggested by Grimm et al. (2020b), discuss the data that is generated by our study (*data mapping*) and the relevant threat actors (*actor mapping*). Afterwards, we present our study design and risk assessment. Finally, we omit the *context analysis* and *validation*, although considered part of threat modeling, as they do not contribute much to our general risk assessment.

### Situation in Afghanistan

After the Taliban regained power in Afghanistan in 2021, they began to search for oppositional and critical journalists. Individuals suspected of opposing the Taliban or their religiously motivated rules are now facing investigation and prosecution. Suspicion can be raised when individuals have ties to *western* contacts or organizations, such as on Facebook/Meta, when they have foreign phone numbers or names in their address book, or if they use apps (e.g., Signal) commonly associated with *western* organizations. In case of identification and detention, the Taliban may use seized phones and computers to identify or locate additional individuals to investigate. In some known cases, Taliban members took over Facebook/Meta accounts and posted incriminating content, presumably in order to fabricate evidence for the prosecution. To locate individuals to be charged and prosecuted, the terror group has used open-source intelligence methods such as *Facebook searches*. They also use physical checkpoints to detain individuals and examine their devices for incriminating content. In addition to the participants’ safety, the safety of related persons, such as family, friends, like-minded journalists, human right defenders as well as journalistic sources are important to consider.

### Data mapping

Before we discuss the data generated by our study, we need to briefly summarize our hypothetical study design: First, we would obtain contact to study participants (Afghan journalists) through contacts recommended by RSF and subsequently use snowball-sampling to find further interlocutors. Should a person agree to being interviewed, we would exchange a consent form for the interview. Then, the interview would be recorded for later processing. For the analysis, each recording would be transcribed, coded, and prepared for publication. With regard to the first part of threat modeling (data mapping), this produces several data artefacts like audio recordings and textual notes, including contact data. Since interlocutors would be contacted via digital means, chat histories would be collected as a byproduct of communication. Should any of this data be compromised, this could result in serious harm for our study participants and others related to the interviewees.

Our hypothetical study design adds two more pieces of information worth protecting: 1) the content of communication, in case it might reveal names or other compromising information and 2) the mere fact that our interlocutor would have taken part in the study. For example, alone the fact of a non-Afghan researcher showing up as a friend in an Afghan’s Facebook/Meta account might already be enough to raise suspicion.

In addition to the data that would be collected, metadata would be generated in other locations, including information such as phone numbers, the number of our interlocutors, the frequency of messages sent and received, including the time and duration of phone calls. The extent of harm that this data could cause varies. Should it be possible to establish a link to a *western* organization, this could lead to serious harm. If it appears inconspicuously, however, then we expect it to cause no harm. Table 2 (see Appendix) provides a summary of these potential harms.

### Actor mapping

The second part of threat modeling deals with actor mapping. Based on the presented situation, the most prominent actors are the Taliban, in the form of government officials, military and police personnel. The Taliban have demonstrated their ability to use open-source intelligence, to seize devices, and to investigate them. The Pakistani government and their intelligence services support the Taliban, making it plausible that the Taliban have limited forms of digital forensic capabilities, communication surveillance, and internet surveillance. It is currently unclear to what extent internet companies such as Facebook/Meta or Apple are cooperating with the Taliban or to what extent such companies refrain from actively preventing their activities on their platforms. Overall, the Taliban and their allies would have an interest in the aforementioned data. The Taliban are recognized as a terrorist organization by the United Nations, making surveillance of our communication with Afghan citizens plausible.

Besides the Taliban, other actors might be interested in this data. Western intelligence services, such as the NSA (National Security Agency) or GCHQ (Government Communications Headquarters), utilize mass surveillance in the form of bulk metadata collection^6^ in order to identify terrorist threats and criminal organizations. Additionally, the communication patterns of our interviews might seem atypical and raise red flags, possibly entailing further investigation. The surveillance capabilities of these actors further include legal investigative methods such as wiretaps and search warrants and the use of spyware to circumvent end-to-end encryption or extract information from devices.

Another threat actor are commercial service providers for communication or online services. These collect usage patterns and interests, mainly in order to improve targeted advertising. They might, however, also sell user data to other companies, or inadvertently expose this information due to software vulnerabilities or insecure server configurations. Another group with a potential interest in such data consists of cyber criminals. These are usually financially motivated and utilize phishing attacks in order to take over accounts, or steal and encrypt files on users’ computers in order to demand a ransom (ransomware).

The final threat actor includes colleagues of ours or other university members with access to the offices or devices where data is stored. In a competitive environment, certain colleagues might have an incentive to compromise other research projects for their personal gain, and others might, e.g., only be interested in stealing expensive equipment. If stolen equipment is subsequently sold, the data on this equipment might be compromised in the process. In summary, Table 1 provides an overview of potential threats and actors involved in our case study.

**Table 1:** Potentially involved actors and their capabilities

Actor	Capabilities and potential threats
Taliban forces	Open-source intelligence
	Seizure of devices
	Digital forensic capabilities
	Communication surveillance
	Internet surveillance
Western intelligence services	Collection of bulk metadata
	Issuing wiretaps and search warrants
	Spyware
Afghan intelligence and police services and allies	Collection of bulk metadata
	Issuing wiretaps and search warrants
	Spyware
Commercial service providers	Collection of usage patterns and interests
	Targeted Advertising
	Selling usage data
	Unintentional disclosure of data
Cyber criminals	Phishing attacks
	Ransomware
Colleagues and other staff	Sabotage of research
	Theft of equipment

Source: own representation

### Final study design

In the following section, we present a detailed description of our final study design.

#### Technical preparation phase

Before contacting our interlocutors, some technical arrangements would need to be made. We would factory-reset a smartphone with a physical audio jack and an up-to-date operating system. The phone would be protected by a PIN code. Furthermore, we would install *WhatsApp* (see Table 8 [Appendix] for justification) and activate two-step verification. For this we would have one of our initial contacts acquire a SIM card with an Afghan prefix, and then forward to us a WhatsApp activation code for this number. Our WhatsApp profile would be set to a commonly used name (e.g., in Dari) without a profile picture.

Furthermore, we would set up a freshly installed computer running a recent Windows operating system with physical audio output and input jacks. The computer would be set up with a local account and the Windows’ built-in encryption tool *Bitlocker*. The computer would be installed with an audio editor, a coding program, and the *VeraCrypt* encryption software, and would be disconnected from the internet after installing all necessary tools. The phone’s audio jacks would be connected to the computer in a way that the computer would be able to record both interviewer and interviewee. Interviews would be stored on this computer in a VeraCrypt container with a randomly generated passphrase and a keyfile that would be stored on a USB pen drive. When not in use, the pen drive would be stored in a different office than the computer.

#### Interview phase

When asking an interlocutor about additional contacts, we would also ask them to assess when the best time would be to reach these contacts to ensure their safety. Before contacting a potential interlocutor, we would set the *disappearing message* timer to 24 hours. In the first message, we would introduce ourselves and ask if they would be interested in participating in our study. If they answered no, we asked them to delete the chat and instructed them on how to do so. If they answered yes, we asked them about the best time to contact them, instructed them on how to delete the chat history, and postponed further communication.

Before each interview, we would send the interlocutor a translated consent-form. Then, at the beginning of the WhatsApp call, we would start the recording using the audio editor *Audacity* and request verbal consent for the interview. After the interview, we would instruct the interlocutor to delete the entire chat and then end the recording.

#### Analysis phase

After the interviews, personal and sensitive information would be deleted from the audio recordings and a manual transcription would be carried out, without being connected to the internet. Once the transcription would be completed, the audio files would be deleted and the transcripts would be coded via the coding software *MAXQDA*.

#### Deleting data

In order to remove any traces of the audio files and transcriptions, we would re-encrypt the computer with a fresh Bitlocker encryption key. Additionally, we would physically destroy (shred) the USB pen drive containing the VeraCrypt keyfile, as the recorded data encrypted by this keyfile are of high protection value. As flash memory (used in USB pen drives and SSDs) is hard to erase via software we consider the option of physical destruction of the storage containing the keyfile as the safest method.

### Risk assessment

Based on the threat actors, their capabilities, and our study design, we identified a list of threat scenarios. For each scenario, we estimate the probability of a threatening event or attack, its success probability, and the harm it would cause. On this basis, we then assess the risk for the presented scenario. An overview of these scenarios can be found in the appendix (see Appendix, Tables 3–6).

Generally, our assessment is based on the following considerations: WhatsApp offers end-to-end encryption, thus protecting communication content. Furthermore, it is commonly used by Afghan civilians, making it not suspicious to use the application. Even if the Taliban were to detect the communication with our interlocutors, we can minimize the harm by using an Afghan phone number and contact name to avoid attention. By utilizing risk-minimizing strategies (disappearing messages, deletion of chat history and contacts) we can further control the overall risk. While we believe that *western* intelligence agencies might detect our communication, we deem it unlikely that they would take any actions that would harm our interlocutors. Finally, by using an offline computer, the chances of success of attacks launched by cyber criminals are negligible.

## What to consider when working with digitally mediated methods

After having presented a concrete hypothetical scenario from Afghanistan, further considerations as well as data-secure and privacy-friendly applications will be presented based on the literature review and the qualitative interviews in order to provide a more comprehensive view of the academic discussion. Our recommendations are intended to show that risk assessments need to be highly individualized and should allow for reflection on the level of security and privacy required for each research phase. Overall, this section seeks to provide researchers with a broad overview of what to consider when working with digital methods (especially surveys and interviews) and to assist them in planning their own research project after having learned about a concrete empirical example. The following recommendations are based on advice provided by security experts and organizations in 2021, potentially changing over time, e.g., due to technological progress. This study neither claims to cover all important aspects, nor to present all existing data secure applications—it rather hopes to reinforce debates within the academic community and to encourage more data secure and ethically justifiable research in the foreseeable future. In addition to specific recommendations, a list of several ethical considerations was created (see Appendix, Table 7).

For better structuring, the following aspects for reflection and presentation of useful tools are divided into different sub-categories, such as first contact and data analysis. Here, we focus on data collection as this is where, we think, the most ambivalence can be seen.

### Getting prepared: risk assessment

As already illustrated (see sections “Security Plan and Threat Modeling” and “Hypothetical case study: Afghanistan (2021)”), prior to initial contact with potential respondents, researchers should consider whether data misuse has previously occurred in the country or context of interest, what risks might be associated with the research, which people are potentially at risk, and who might be potential adversaries (IP1)^7^. In some contexts, this may be obvious, in others it may be very difficult to assess simply because many aspects from the digital space are not publicly available (IP8). In case a risk analysis indicates that people could be observed or wiretapped, it should be reconsidered whether a study can be carried out any further (IP6). According to Grimm et al. (2020, p. 3), “there is no shame in valuing your own or your team’s physical and mental well-being over ‘sexy’ research.” Generally, it is advisable to talk to others about the research intent and to reflect critically about potential risks before starting or continuing the study (IP8). Concerns can be summarized in a risk matrix, listing the risk probability of a potential treat and potential impacts (Grimm et al. 2020a). In our case study, we deemed the remaining risks associated with the study acceptable (see section“Actor mapping”).

### First contact

According to IP8, access to participants is one of the biggest challenges in research: “Security [related] and ethical questions come with access. It is a huge responsibility that we, as researchers, have when we engage with participants (…)—especially with higher risk groups.” Most commonly, the first contact between the researcher and the study participants is initiated by the researcher. Hence, the researcher sets the direction in terms of the medium to be used and is usually more familiar with the software (IP1). The instructions given by the researcher might create a feeling among study participants that their own decision-making power is being overruled and that they are not trusted with assessing their own risks. This position of power and the fact that researchers usually hold a higher social position should be critically reflected—in any research step in both analog and digital research (Dell et al. 2012; Fujii 2012; Cronin-Furman and Lake 2018). In this context, it should be considered that people also use less data secure applications for their own private communication (IP8). Here, a balance between self-agency (Markham and Buchanan 2015) and data security is important. Interviewee 4 stated that “you have to pick people up where they are and take the most secure of the platforms they use.” Applications that interlocutors may not be familiar with and thus use incorrectly should generally not be used (IP5).

Often, the first contact in formal contexts occurs via e‑mail. So far, it seems that most people do not use *ProtonMail* (IP5) or public-key cryptography, such as *Pretty Good Privacy* (PGP), to end-to-end encrypt^8^ e‑mail content, leaving it vulnerable to spying. When first contact is made via e‑mail, the first unencrypted message should not contain sensitive information. Attaching one’s own public key to the e‑mail demonstrates a willingness for encrypted communication (IP8), as does publishing the key, e.g., on a personal website or public PGP key server. Unfortunately, experts we interviewed said that many people do not know how to use PGP properly (IP4), which is also stated by Grimm et al. (2020c). Phone calls and text messages (SMS) likewise suffer from security gaps and are nevertheless frequently used as a means of contact and survey tool. In very sensitive contexts, it is advisable to avoid insecure options or to switch immediately to more data secure messengers. In some cases, this seems almost impossible due to continuing contact restrictions (IP11) and the fact that, particularly in rural areas of some countries, many people lack access to (high-speed) internet. Telephony may thus be the only way to contact and interview people (IP9).

Another approach of recruiting study participants is to disseminate a project description including multiple contact details in public social media groups, including through existing contacts (gatekeeper) or organizations (IP5; IP8). Generally, the selection of groups should be well considered (as groups may be surveilled). Potentially interested participants may then decide whether they wish to participate and if so, how to contact the researchers. In this procedure, care should be taken to avoid pressure by, for example, the forwarding entity and ensure that participation is completely voluntary (IP3). IP8 mentioned that even though they are aware of many cultural and safety-related issues, they are “never as aware as a local gatekeeper.” Therefore, it is important to rely on their recommendations (IP8).

### Data collection

During data collection, a number of considerations need to be taken into account, which is why we describe them in more detail. Here, recommendation for specific data-secure applications is less simple and unambiguous than for other research phases, given the fact that *human* security does not necessarily correspond to *data* security. In some cases, as the example of Afghanistan has clearly illustrated, less data-secure applications such as WhatsApp may be more secure for interlocutors on account of other circumstances.

Numerous organizations or institutional review boards (IRBs) at universities seem to prescribe that the communication software to be used shall address legal constraints such as compliance with the GDPR, without providing concrete recommendations (IP8; IP10). Consequently, decisions usually need to be made individually without receiving proposals beforehand. Commonly, it is recommended to not connect research computers to unfamiliar networks like public Wi-Fi, and to use a trustworthy VPN (*virtual private network*)^9^ when unavoidable. Certain situations and contexts require that researchers anonymize their internet traffic. In these cases, routing the complete network traffic via *Tor* should be considered, which is possible, for example, through *The Amnesic Incognito Live System* (Tails 2021). Generally, the technical design of Tor makes it unsuitable for telephony (IP4). Recognizing that some applications are more technically demanding, International Alert (2021) emphasizes the importance of ensuring that not only digitally experienced people are reached but that persons of diverse backgrounds and identities are included.

If an interview or a written survey is planned, principles for secure communication (CISA 2020), encrypted messenger services, and secure video conferencing offer suitable options for data collection.

#### Instant messengers

Instant messengers are crucial for everyday communication as they provide users with numerous attractive features. However, they often neglect privacy functions. A general overview of which instant messengers meet certain criteria related to data security and privacy can be found in the appendix (see Appendix, Table 8). In the following, we elaborate on the direct impact on certain privacy aspects that may need to be considered. The most evident risk seems to be the need for a phone number to register for a messenger. This could pose a problem for people who cannot or are not willing to share their phone number. Here, the question of how a messenger exchanges data with other applications must also be considered, as it touches upon similar privacy related issues. For example, information from the address book should not be uploaded to hide the own social network as well as prevent the association of names to phone numbers from getting leaked. Also, exchanged media files should not be automatically saved in the phone’s gallery, in case a third party has physical access to the phone and has a look at the gallery, or there is any synchronization between the phone’s gallery and a cloud storage. Another useful feature is self-deleting messages, which can be an additional measure to prevent disclosing exchanged information in situations like physical access to a phone by a third party. To protect chat histories and alike, an app access protection, such as *passcode/pin or fingerprint lock* can be useful. As metadata can be used to deduce communication insights, it is generally helpful for a messenger to store as *little metadata *as possible or to avoid the generation of metadata during use. Not capturing and forwarding the online status can be an additional feature to prevent inference of identity.

Data security aspects go hand in hand with privacy protection aspects. Modern messengers must implement strong and established encryption, in the form of *end-to-end encryption* (E2EE). That also requires the app to store data only locally and encrypted. For non-peer-to-peer messengers, it would also be beneficial to use *multi-factor authentication* to present a combination of two or more credentials to verify a user’s identity for login, e.g., by a combination of password and PIN code. In this way, breaking into an account is made more difficult.

It is important for a messenger service to be trustworthy and user-friendly. Being an *open-source* software (meaning both client and server code) allows for independent auditing of important properties of a messenger, but is not a guarantee for error-free or ethical-correct software. In case of centralized messengers^10^, e.g., Signal or Wire, additional considerations regarding the server infrastructure are helpful. In the best case, centralized messengers rely on trustworthy infrastructure hosting that is well described (transparent) and takes measures to ensure privacy, e.g., preventing storage of connection details. When messenger maintainers are transparent regarding their financial funding and cost, users can determine which parties might monetize their data. Transparency about the data collected as well as transparency about pseudonymized or anonymized data are additional trust measures. Aspects of accessibility and compatibility should also be considered, which lower the barriers to using a messenger. Availability in numerous languages and being free of charge are important aspects, for example.

The following aspects should be considered when researchers plan to propose a service that is less popular (e.g., Signal). Firstly, rarely used applications could arouse suspicion and create the impression that the person has something to hide (IP5). During mobile phone inspections, for example, this can lead to questioning or arrest in certain cases. Secondly, not all operating systems support such applications. It should be examined in advance which operating systems are mostly used in the respective country and which applications can then be used. Regardless of the messenger used, all messages and exchanged numbers should be deleted from all devices at the end of the research (IP4).

#### Video conferencing platforms

Many aspects to consider when choosing a messenger also apply when choosing a video conferencing tool. Concerning privacy aspects, it is beneficial if a video conferencing system does not require an account or registration. In order to prevent the unintentional sharing of the webcam or microphone before joining the video conference, a lobby screen to setup video and audio settings should be provided so that it is possible to adjust the desired settings before entering the conference room (CISA 2020). As is the case for messengers, it is generally helpful if a video conferencing software stores as little metadata as possible. Aspects regarding data security, trust and user-friendliness, and accessibility and compatibility are similar to messengers. A difference is that video conferencing software might be used ad-hoc via a link without the need for an account, and is accessible via modern web browsers without additional software. An overview of which video conferencing tools meet criteria related to data security and privacy can be found in the appendix (see Appendix, Table 9).

#### Digital questionnaires

Digital survey tools are used to conduct both qualitative and quantitative studies. When selecting a survey tool, attention should be paid to what, where, and how data is stored, and for which duration. *SoSci, KoBotoolbox* (IP9), *Akvo* (IP10), *SurveySparrow, LimeSurvey*, and *Survey Monkey* are professional tools to conduct online surveys and comply with ethical approaches. They are known differently in certain regions and thus also follow differing legal regulations such as the GDPR (SurveyMonkey 2017; SoSci 2021).

### Data analysis

Depending on the type of data collection, different steps are required for data analysis. In case of interviews, the conducted interviews are mostly transcribed either manually or automatically using software. Manual transcription can be done offline and without uploading audio recording to the software manufacturer, while tools for automatic transcription often require the use of an online transcription service. Generally, in very sensitive contexts, such as with our hypothetical case study, it seems appropriate to only transcribe manually (i.e., offline) to avoid further technical misuse and privacy violations. When transcribing manually, personal attributes should be anonymized or pseudonymized to ensure data protection and to maintain study participants’ trust (IP5). Certainly, manual transcription consumes more resources—it is significantly more time-consuming and therefore more cost-intensive. Generally, the transcribed documents need to be saved. Instructions for data storage will be given in the section “Data storage”. Once transcription is completed and the quality of the transcript verified, audio files should be deleted.

In case a (semi-)automatic transcription software such as *Amberscript* is used, the software should be GDPR compliant since sensitive audio data will be uploaded to a server (Amberscript 2022). Generally, it is advisable to cut out sensitive personal data from the audio file before uploading it to prevent potential traceability. Transcripts are often coded using software such as *Nvivo, ATLAS.ti*, or *MAXQDA*. Similar to the transcription software, attention should be paid to ensure that online applications are GDPR compliant, that data is encrypted, and deleted from the servers after a short period of time (Atlast.ti 2016; Nvivo 2022).

### Data storage

In light of the increasing number of data breaches, different types of data need to be protected to varying degrees (IP6). Classification, relying on three main characteristics (access control, content, and storage), can be used to assign data to different protection levels. Most sensitive data (e.g., personal information) which may pose risks if stolen need to be best protected, i.e., by good storage-encryption, orderly key management, and limited access on a need-to-know basis. Researchers should 1) ensure their own devices are safeguarded, 2) choose appropriate data storages, and 3) delete research data after utilization:

1. *Safeguarding devices*: To get a basic level of device security, we recommend regular software updates, a firewall blocking undesired traffic, strict fulfillment of one’s own organizational security policies (like not attaching foreign hardware, e.g., USB media), and preventing any installation of private software. The use of *anti-virus software* is ambivalent to some extent: As the idea of scanning files for malicious code is seen useful, the anti-virus software itself could be target of sophisticated attacks (Wressnegger et al. 2017). One consideration is to just use on-demand anti-virus scanners on separate computers for scanning retrieved files and media storages prior to opening it. Depending on the threat model, wiping and reinstalling devices before and after a study might be useful.
2. Appropriate *data storage locations*: Generally, three options of data storage locations may be considered: 1) encrypted data storage on a personal computer, 2) data storage on portable devices such as external hard drives (encrypted by tools such as VeraCrypt [IP6]), and 3) data storage on a cloud service that offers hosting data on a remote server accessible at any time via the internet. Storage on a cloud service should only be considered if multiple parties at different locations work with the study data. When selecting a cloud service, such as *Sync.com* and *pCloud*, attention should be paid to the respective data protection policies (pCloud 2022; Sync.com 2022) and the stored data must be encrypted by tools, such as *boxcryptor *(Secomba GmbH 2021). If data is stored, for example, in a Word document or Excel file, a secure passphrase, consisting of a combination of words (e.g., *diceware*), can serve as an additional protection. Generally, access to confidential data should be limited to internal project partners (Shaikh and Sasikumar 2015; Security.org 2018). Communication about the protection goals and minimum standards with all persons with access to the data is important.
3. *Deleting research data*: Deleting data can be difficult. When research data was always encrypted, re-encrypting the entire storage device with a new encryption key should suffice. In order to erase unencrypted storage devices, we advise to consult the manufacturer of the device.

As already mentioned, regarding our case study, we would protect stored data by encrypted storage on a computer (Grimm et al. 2020c). As we would classify the audio files and transcripts to be worth of high protection, we would save those files only in encrypted VeraCrypt volumes. The volumes would be protected by a keyfile, which would be stored on a separate USB pen drive. The USB pen drive would be kept at a different location than the encrypted container—implementing an additional form of physical access control.

### Data publication

According to Lowenberg and Puebla (2022, p. 3) it is important “before the research begins (…) to learn (…) what it means to publish data.” In general, it should be ensured that study participants’ personal data are completely *anonymized* or *pseudonymized *(Fujii 2012). As Henry et al. (2022) elaborate, how third parties use publicized data in the future is not in control of the researchers, and may not be foreseen. Therefore, the authors argue for critical reflection on the release of the amount of data, to publish only the data that is really necessary. Here, special care should be taken to avoid identifying individuals by naming specific statements and characteristics. Samarati and Sweeney (1998) observed that datasets comprising anonymized data can be linked together when entries uniquely share certain attributes. By linking, it is possible to identify individuals or attributes about individuals. To prevent data items from being linked, they suggest the notion of *k‑anonymity*, which was later improved by *t‑closeness* (Ninghui et al. 2007) and *l‑diversity* (Aggarwal and Yu 2008). An even more involved solution offers *eps-differential privacy*, which promises to quantify the privacy loss an individual suffers from being included in a dataset (Dwork 2006).

## Discussion and conclusion: secure research with digitally mediated methods

In times of digitalization and the COVID-19 pandemic, digital communication is becoming increasingly important—including in research. In addition to existing challenges of physical research (such as safety, traumatization, and logistical difficulties [Moss et al. 2019]), other risks arise in the digital age such as data misuse and digital surveillance. Various examples of e.g., spyware attacks, subsequently implying physical risks, illustrate the overlapping of the analog and digital space (Jones 2022).

So far, there has been little debate about research ethics, data security, and privacy violations during empirical studies in the social sciences. To take on responsibility and protect involved study participants, risks should be identified in advance and, consequently, appropriate security precautions should be taken regarding digitally mediated empirical research (Tolich and Tumilty 2021). Concerning this matter, approaches such as threat modeling, ethical field research, internet research ethics as well as certain critical aspects, such as privacy-related issues and self-critical questioning require careful consideration (Buchanan and Ess 2009; Fujii 2012). Although most researchers try to meet these needs, sometimes mistakes may occur because of overlooking certain (cultural, political) aspects (Fujii 2012). Reality shows that it is difficult to consider and prevail all ethical and secure approaches due to the lack of specific expertise and the striking discrepancy between usability, security, privacy, and dissemination (IP4). In general, when selecting a communication tool during research, it seems that compromises must be made in at least one of these categories. It should be made clear that to date no single communication software exists that is at the same time widespread, user-friendly, and that addresses all security-related issues. Nevertheless, some applications and programs put clear emphasis on data security, such as strong encryption, and have gained popularity in recent years. In general, some scientists (particularly in the social sciences) seem to have little IT security knowledge (Moßbrucker 2020) and the fast pace and complexity of technology development complicate many researchers’ ability to maintain an overview of existing applications and their associated risks. It will certainly take some time until a broad audience shows willingness to engage with these issues, well-known applications become less popular, and user bases are less fragmented. We consider it important that researchers (especially those operating in sensitive contexts) discuss the aforementioned considerations with colleagues and draft reality-based guidelines that may be adjusted depending on the situation, having the needs and autonomy of study participants in mind (IP4; IP5). Over time, this will make it easier to assess risks and use initially complicated looking applications.

As outlined in the paper, decisions regarding ethical concerns and privacy are highly situational and circumstances may change very quickly (IP5). This becomes evident in the case of Afghanistan, where the safety conditions for a majority of the country’s population have changed drastically since the Taliban seized power in August 2021. Since no universal recommendation for secure data collection can be made and there is no *one fits all solution*, it is advisable to carry out an individual risk analysis/threat model for each planned study, similar to what we have presented here (Electronic Frontier Foundation 2021b). Aiming to address as many safety-relevant aspects as possible and to provide a holistic analysis of each research project, we advise to combine concepts from various disciplines such as peace and conflict studies with theoretical approaches from IT security (such as threat modeling) (Kenneally and Dittrich 2014; Lauber-Rönsberg 2018; Tolich and Tumilty 2021). Aspects such as data and actor mapping, context analysis, and validation should be incorporated in any case.

In summary, digitally mediated research methods promote novel approaches in the social sciences that require ethical and privacy guidelines appropriate to these new circumstances (Thompson et al. 2021). It is essential to monitor new technical developments and to reconcile different realities of life. This implies, amongst others, that human security does not necessarily go hand in hand with data security (for example in our case study), that different applications are used around the globe, and that different risks are associated with each application and research procedure. Generally, more empirical examples are needed in academia to illustrate the practical implementation of existing theoretical concepts, such as threat modeling, and to further clarify that each context shall be evaluated differently. The fact that each research procedure shall be assessed individually indicates that currently often existing requirements, such as the GDPR, are not applicable to all cases. Our specific case study clearly shows that, in the example of Afghanistan, a less data secure and non-GDPR-compliant instant messenger (WhatsApp) seems the most appropriate for contacting potential interlocutors and for conducting research, for the reasons explained in the sections “Hypothetical case study: Afghanistan (2021)” and “What to consider when working with digitally mediated methods”. Our paper seeks to demonstrate that it is vital to be thoroughly familiar with one’s own research context to adequately assess potential risks. In conclusion, we wish to stimulate a sustained debate on research ethics and encourage researchers to deal intensively with threat modeling and technical applications, while losing the apprehension of making major mistakes. Following Mwambari et al. (2021, p. 2), we believe that every mistake and “crisis presents an opportunity to re-think research practice.”

## Limitations

Our threat model has been developed with utmost conscience of the circumstances in Afghanistan since summer 2021. At any moment, the socio-political situation may change, new security bugs may be discovered, or new applications developed, meaning that this paper can only reflect on the status-quo and on one specific country. Furthermore, we cannot provide a general recommendation for the conduction of digital research applicable across all scenarios, as use cases vary greatly depending on the context. While the literature review captures a broader range of opinions and assessments from around the world, the interviews can only cover individual experiences, illustrating a non-representative excerpt of reality. Further studies may complement these insights, capturing more voices from multiple contexts and further highlighting how circumstances may change over time.

## Appendix

**Table 2:** Gathered data and their risk assessment

Type of Data	Harm when compromised
Audio recordings, transcripts, consent forms, textual notes, chat histories	Serious harm
Call and chat metadata	Serious or no harm

Source: Own representation

**Table 3:** Threat from Taliban or allies

Threat from Taliban or Allies	Probability Attack	Probability Success	Harm	Risk
Discover comm.: bulk data collection	Low	Low	Low	Low
Discover comm.: on phone after initial contact, before interview	Medium	Low	Low	Low
Discover comm.: during interview	Low	Low	High	Low
Discover comm.: after interview	Medium	Medium	Low	Low
Break WhatsApp encryption	Low	Low	High	Low
Spyware already on interlocutor phone	Low	High	High	Medium
Spyware attack on researcher	Low	Medium	High	Low

Source: Own representation

**Table 4:** Threat from cyber criminals

Threat from Cyber Criminals	Probability Attack	Probability Success	Harm	Risk
Phishing attack on WhatsApp	Medium	Low	High	Low
Ransomware attack on Computer	High	Low	High	Low

Source: Own representation

**Table 5:** Threat from western intelligence agencies

Threat from Western Intelligence Agencies	Probability Attack	Probability Success	Harm	Risk
Discover comm.: bulk data collection	High	High	Low	Low
Wiretaps and search warrants	Low	High	Low	Low
Spyware	Low	Medium	Low	Low

Source: Own representation

**Table 6:** Threat from WhatsApp

Threat from WhatsApp	Probability Attack	Probability Success	Harm	Risk
Collect usage data and interests	High	High	Low	Low
Sell/Share usage data	High	High	Low	Low
Inadvertent exposure of data	Low	High	High	Medium

Source: Own representation

**Table 7:** Aspects for consideration when conducting research, using digitally mediated methods. If the risks analysis indicates a highly sensitive context with a potential risk of surveillance, minimum IT security standards, as described above, should be applied

Phase	Aspects to consider
*Concretization of the project*	What do I want to achieve with my research project? Is my research related to human research and thus involves ethical issues? If this is the case, have I submitted a request to an institutional review board (IRB)?
	Do Data Protection Regulations (e.g., GDPR) affect my research?
	Do I understand the needs and interests of those potentially participating in my study? What are common/opposing interests and what is the cultural context?
	Who will benefit from this study and in what ways?
	What are my personal biases (e.g., gender, race, ethnicity)?
	Have I critically reflected that my subjective perspective plays an essential role for implementing the study and that it is difficult to analyze unfamiliar facts as a member of a certain social group?
	How do I deal with the fact that I am mostly an *outsider* from potentially interviewed groups?
*Conflict analysis*	What is the root cause of the crisis?
	What types of data do I produce or make use of during my research? What information do I collect, store, or communicate?
	Who is involved in the crisis? How do these parties relate to each other?
	What are the different reasons why parties participate in the crisis? What do different parties want to achieve?
	Are acts of violence and cases of digital surveillance known?
	Who might be interested in my data, the sources of my data, and in its further use?
	Who would be affected by data breaches?
	What software and ICT services do I rely on during my research?
	What level of digital proficiency do I and/or my research team possess?
	Who else might have access to my computer?
	Should I pay special attention to my protection?
	Can I use a SIM card registered in my name?
	Have I renamed or deleted sensitive contacts on my end device?
	How likely is it that I will need to protect it?
	How much trouble am I willing to go through to try to prevent potential consequences?
	How bad are the consequences if I fail?
*First contact*	What is my familiarity with local customs?
	Are all study participants of legal age?
	Do I make contact via data secure communication tools?
	Do I make sure the person is voluntarily participating in my study? How do I intend to obtain informed consent? Is the participant aware that consent can be withdrawn?
	How do I ensure that all participants understand the purpose of the study, potential consequences associated with the authorization and that all have enough time to think about their choices?
	How do I inform illiterate study participants?
	Have I considered whether participants are financially rewarded? If so, how can it be ensured that participation is not based solely on financial incentives, that the individual’s decision to participate is not distorted and risks thereby underestimated?
	Do participants need to be provided with equipment such as SIM cards in order to participate in the study?
	Are the participants confident with the equipment, tools, software or is help needed?
*Data collection*	*Online Questionnaire* Is the survey translated into the language that the participants speak, ensuring that they can understand everything correctly? Is it assured that the translation accurately depicts the study information? Is a translator needed? Can questions be skipped in case people do not want to answer them? In case of an online survey, is the data stored anonymously on the server? Is an inclusive and safe online space created?
	*Online Interview* Is the person to be interviewed alone and/or how can privacy be provided? Is it acceptable for the person that the interview might be recorded? Is the tool we use to record data secure? Is the questionnaire translated into the language that the participants speak, ensuring that they can understand everything correctly? Is all the data I collect truly important to my research project or can certain aspects, especially biometric data, be left out (principle of data minimization)? Is all my data pseudonymized or anonymized? Can tracking be possible despite pseudonymization or anonymization? If so, what information can I leave out so that this is not the case? Can questions be skipped in case people do not want to answer them? Have I informed and advised the interviewees to delete all survey-related data/phone numbers from their device?
*Data analysis*	Are the tools I use for data analysis secure regarding privacy?
	Do I know where the servers are located and if the company stores the data or deletes it after the study is completed?
	In case I share data with colleagues (e.g., for transcription), have I made sure that they also pay attention to data security on their end devices and do not misuse the data?
*Data storage*	Do I store my data in a secure location so that no one can access it?
	Is all of my data encrypted?
	Are my computer, documents, and cloud containing sensitive data, password protected? Is my password as secure as possible?
	Am I aware of potentially relevant data protection legislation?
	Have I cut out personal information from the audio files so that individuals can no longer be identified via the audio files?
	Do I keep the audio files, transcripts, and consent forms in different locations?
*Publication*	Is all data anonymized/pseudonymized and guaranteed that the published information cannot be traced back to an individual?
	Ensure, in case you want to make your data FAIR (findable, accessible, interoperable, reusable), that no sensitive information will be published. If data is very sensitive, it is recommended not to publish it in a public repository

Source: own representation, building, amongst others, on ideas from Dawson (2020), the EFF (2021a), Grimm et al. (2020a, b, c), and Jeong (2008)

**Table 8:** Properties of messaging tools that need to be scrutinized, with example apps. On the basis of the OSI security architecture (Verschuren et al. 1993) and the reviewed literature (amongst others, Oppliger 2014; ITGovernance 2021), a number of criteria for secure communication software were derived in order to evaluate numerous apps

		Signal	Wire	Briar	WhatsApp	Telegram	Threema	Viber	Wechat
Privacy	Stores as little metadata as possible or avoids metadata during use	✓	✗	✓	✗	✗	✓	✗	✗
	Phone numbers are not required for registration	✗	✓	✓	✗	✗	✓	✗	✗
	Self-deleting message service provided	✓	✓	✗	✓	✗^d^	✗	✓	✗
	Information from address book is not uploaded	✗^a^	✓	✓	✗	✗	✗^a^	✗	✗
	Does not capture and forward online status	✓	✗	✗	✓	✗	✓	✓	✓
	Provides the opportunity of being found by nicknames rather than by phone number	✗	✓	✓	✗	✓	✓	✗	✓
	Provides the possibility of a passcode/fingerprint lock	✓	✓	✓	✓	✓	✓	✓	✗
	Does not automatically save received media to the gallery	✓	✓	✓	✗^b^	✓	✓	✗^b^	✓
Data security	Uses strong encryption and comprehensible cryptography	✓	✓	✓	✗	✗	✓	✓	✗
	Supports multi-factor authentication	✓	✗	✗	✓	✓	✓	✗	✗
	Only stores data locally and encrypted	✗	✗	✓	✗	✗	✓	✓	✗
	Provides security against a strong attacker/threat model	✓	✓	✓	✗	✗	✓	✓	✗
Trust and user-friendliness	Is open-source, provides communication protocol and source code	✓	✓	✓	✗	✓	✓	✗	✗
	Provides a transparent and trustworthy infrastructure hosting	✗	✗	✓	✗	✗	✓	?	✗
	Is transparent regarding its financial funding and costs	✓	✗	✓	✗	✗^c^	✓	✓	✗
	Let the user know which data is collected and stored	✓	✓	✓	✗	✗	✓	✗	✗
	Ensures anonymized or pseudonymized data	✓	✓	✓	✗	✗	✓	✓	✗
	Accessible security audits are conducted by independent third parties	✓	✓	✓	✗	✓	✓	✗	✗
Accessibility and compatibility	Is applicable on numerous operating systems	✓	✓	✓	✓	✓	✓	✓	✓
	Is free of charge	✓	✓	✓	✓	✓	✗	✓	✓
	Is available in numerous languages and app stores	✓	✓	✓	✓	✓	✓	✓	✓
	Is GDPR compliant (as an example of a legal restriction for performing studies)	✗	✓	?	✗	✗	✓	✓	✗

Source: own representation

^a^Uploads hashes of phone numbers from address book

^b^Can be disabled

^c^Until 2021 financed by founder

^d^In secret chats

**Table 9:** Properties of video conferencing tools that need to be scrutinized, with exemplary apps

		Jitsi Meet	Big Blue Button	Zoom	Skype	Microsoft Teams	Cisco Webex
Privacy	Stores as little metadata as possible or avoids metadata during use	✓	✓	✗	✗	✗	✗
	An account/registration is not required	✓	✓	✓^c^	✓^c^	✓^c^	✓^c^
	Provides lobby to setup video/audio (e.g., disable webcam)	✓	✓	✓	✗	✓	✓
	Allows for rooms to be password-protected	✓	✓	✓	✗	✗	✓
Data Security	Uses strong encryption and comprehensible cryptography	✓	✗	✓	✓	✓	✓
	Only stores data locally and encrypted	✓	✗	✗	✗	✗	✗
	Provides security against a strong attacker/threat model	✓	✗	✓	✓	✓	✓
Trust and user-friendliness	Is open-source, provides communication protocol and source code	✓	✓	✗	✗	✗	✗
	Provides a transparent and trustworthy infrastructure hosting	✓*	✓*	✗	✗	✗	✗
	Is transparent regarding its financial funding and costs	✗	✗	✓	✓	✓	✓
	Accessible security audits are conducted by independent third parties	✗	✗	✗	✗	✗	✗
Accessibility and compatibility	Works in browser, without additional software	✓	✓	✓	✓	✓	✓
	Works on smartphones	✓	✓	✓	✓	✓	✓
	Is free of charge	✓	✓	✓^b^	✓^b^	✓^b^	✓^b^
	Is available in numerous languages	✓	✓	✓	✓	✓	✓
	Is GDPR compliant (an example of a legal restriction for performing studies)	✓^a^	✓^a^	✗	✗	✗	✗

Source: own representation

^a^Relies on server operator

^b^There is a paid version

^c^Only the host needs an account

## Footnotes

1 Privacy concerns responsible data handling, while security involves protecting data from malicious threats (Tawalbeh et al. 2020).

2 The authors of this paper were not involved in the planning or creation of this Digital Care Guide.

3 General Data Protection Regulation. Compliance with the GDPR means that they adhere to the basics of data protection, meaning that, for example, data collected is archived after a maximum of three months and the duration of time spent on the website is not tracked.

6 In bulk metadata collection, actors collect the metadata (who communicated with whom and when, etc.) of all communication in a network. This is usually done with the help of the service provider, for example, a phone company.

7 Interview partner 1 = IP1 and so on.

8 Further information about end-to-end encryption: EFF (2020): Communicating with others. Online available at: https://ssd.eff.org/en/module/communicating-others.

9 Further information about VPN: EFF (2019): Choosing the VPN that’s right for you. Online available at: https://ssd.eff.org/en/module/choosing-vpn-thats-right-you.

10 A centralized messenger needs to connect to servers of the messenger operator for data exchange, even in case two persons chatting are standing next to each other. In contrast, decentralized messengers (also called peer-to-peer messengers) allow the exchange directly between two peers. As the centralized pattern allows for more convenience functions, such as sending a message to a person, that is currently offline, centralized messengers are more common.
